# Graduates of Rush Medical College

**Published:** 1848

**Authors:** 


### ARTICLE IV

### LIST OF THE GRADUATES OF RUSH MEDICAL COLLEGE FOR 1847–'48, WITH THE TITLE OF INAUGURAL THESES.

Camerer, Daniel M., Illinois—On Inflammation.

Chamberlain, William, Ills.—Morbid appearance of the countenance.

Clarke, Joseph A., Wisconsin—On Fever.

Crawford, Alexander B., Mich.—Qualities essential to the Student and Graduate of Medicine.

Darnall, Milton D., Ills.—The Buffy-coat of the Blood.

Golliday, Uri P., Ills.—On the varied forms of Fever.

Hawley, Reuben S., Mich.—The Physiological Process of Parturition.

Hobbs, James C. H., Ills.—Podophyllum Peltatum.

Hough, Erastus G., Ills.—Disguised forms of Fever.

Huey, George J., Ills.—Intermittent Fever occurring in the U. S. Army in Mexico.

Jones, Ambrose, Wis.—Medical Topography.

Knott, Christopher W., Ills.—On Disease of the Eyes.

Lovejoy, J. Collins, Ind.—Emetics.

Loftin, Sample, Ind.—Ergot.

Mathews, William, Ind.—The vis medicatrix Natura.

Moor Thomas C., Ind.—Relation of Remedial Agents to General and Special Pathological Conditions.

McGirr, John E., Pa.—Counter Irritation.

McNutt, James H., Ind.—Miasmata.

Newton, John P., Ills.—Emetics.

Nutt, John, Ind.—Entozoa.

Otis, Orvin C., Wis.—Symptomatology.

Osborne, J. George, Ind.—Menstruation.

Pearson, Jonathan, Ills.,—Pneumonia.

Reynolds, Albert J., Ills.—Phthisis Pulmonalis,

Sedgwick, Westel W., Ills.—Billious Remittant Fever.

Sweetland, Warren M., Ills.—Hepatitis.

Stone, Reuben R., Ills.—Nature and Designation of the Food of Animals.

Tucker, James P., Ind.—Intermittant Fever.

Ware, Charles, Wis.—Uterine Hemorrhæge as a complication of Labor.

Warner, C. C., Wis.—Counter Irritants and Counter Irritation.

Warren, Luke W., Ills.—Uterine Hemorrhage.

Whole No.—31.

